# Spontaneous intramedullary abscesses caused by *Streptococcus anginosus*: two case reports and review of the literature

**DOI:** 10.1186/s12879-022-07099-7

**Published:** 2022-02-10

**Authors:** Christian D. Cerecedo-Lopez, Joshua D. Bernstock, Adam A. Dmytriw, Jason A. Chen, Joshua I. Chalif, Saksham Gupta, Joseph Driver, Kevin Huang, Susan E. Stanley, Jonathan Z. Li, John Chi, Yi Lu

**Affiliations:** 1 Department of Neurosurgery, Brigham and Women’s Hospital, Harvard Medical School, 75 Francis Street, Boston, 02120 MA USA; 2 Department of Neuroradiology, Brigham and Women’s Hospital, Harvard Medical School, MA Boston, USA; 3 Department of Medicine, Division of Infectious Diseases, Brigham and Women’s Hospital, Harvard Medical School, Boston, MA USA

**Keywords:** Intramedullary, Abscess, Spine, spinal cord injury, Myelopathy, *Streptococcus anginosus*

## Abstract

**Background:**

Intramedullary abscesses are rare infections of the spinal cord. Intramedullary abscesses often have a complex presentation, making a high index of suspicion essential for prompt diagnosis and management.

**Case presentation:**

We present two cases of intramedullary abscesses referred to and ultimately managed at our institution. Delayed diagnosis occurred in both instances due to the rarity of intramedullary abscesses and their propensity to mimic other pathologies. For both patients, prompt surgical management and the rapid institution of broad-spectrum antibiotics were critical in preventing further neurological decline.

**Conclusions:**

Although rare, it is critical to consider intramedullary abscesses on the differential for any MRI lesions that are hyperintense on T2 and peripherally enhancing on T1 post-contrast sequences, as even short delays in treatment can lead to severe neurological damage.

## Background

Intramedullary abscesses are rare infections of the spinal cord. Having a high index of suspicion is essential for prompt diagnosis and effective clinical management. While intramedullary abscesses are thought to result from the secondary spread of an ongoing infection, often no other primary source of infection can be found [1–8]. Many organisms have been associated with intramedullary abscesses, including *Mycobacterium tuberculosis* in the developing world and gram-positive cocci—i.e., primarily oral or skin flora—in the developed world [9–11].

Here, we report two cases of intramedullary abscesses treated at our institution where no primary site of infection could be identified; of note, both cases were caused by *Streptococcus anginosus*, a species of viridans-group *Streptococcus*. Both patients initially presented with back or neck pain with subsequent, rapidly progressive myelopathy. Both were treated surgically via laminectomies and myelotomies for decompression and abscess drainage/washout, followed by an extended course of broad-spectrum antibiotics. Interestingly, magnetic resonance imaging (MRI) revealed lesions in one of the patients who possessed notable flow voids within the intradural spaces mimicking a spinal vascular malformation.

## Case presentation

### Case 1

A 65-year-old female with a history of hyperlipidemia and osteoporosis was transferred to our institution from an outside hospital with progressive right upper extremity weakness, difficulty ambulating, and a rapidly growing mass in her cervical spinal cord.

Ten days before her admission, the patient developed mild intermittent neck pain that ultimately became constant and severe. Five days later, she developed paresthesias in her hands and feet, which progressed to her mid-thighs and mid-abdomen. Her review of systems was negative for constitutional symptoms such as fevers, chills, or night sweats. As such, she presented to another institution, where an MRI revealed cord edema from C1–T1 and an ~ 8 mm, nodular, intramedullary lesion at the level of C4–5, initially thought to represent an intramedullary tumor. A lumbar puncture revealed elevated protein at 120 mg/dL, glucose at 61 mg/dL (CSF:serum glucose ratio 0.52), and 28 white blood cells (WBC) per µL (with a predominance of PMNs 39%). The patient did not have other notable signs of an active infection (e.g., she was afebrile and with normal peripheral WBC [8.5/µL], high-sensitivity C-reactive protein [hsCRP 1.5 mg/L] and erythrocyte sediment rate [ESR 9 mm/h]), a preliminary diagnosis of neoplasm vs. demyelinating disorder was made, and she was started on high-dose methylprednisolone for 5 days. On hospital day 4, a repeat MRI revealed an increased size of the previously noted lesion to ~ 14 mm and a significant amount of surrounding edema. The likelihood of a neoplastic process was deemed low, and the patient was started on intravenous immunoglobulin and prednisone. Two days later, the patient’s right-sided hemiparesis worsened to the point she was no longer able to ambulate. At that time, the patient was transferred to our institution for definitive management.

**Fig. 1:**
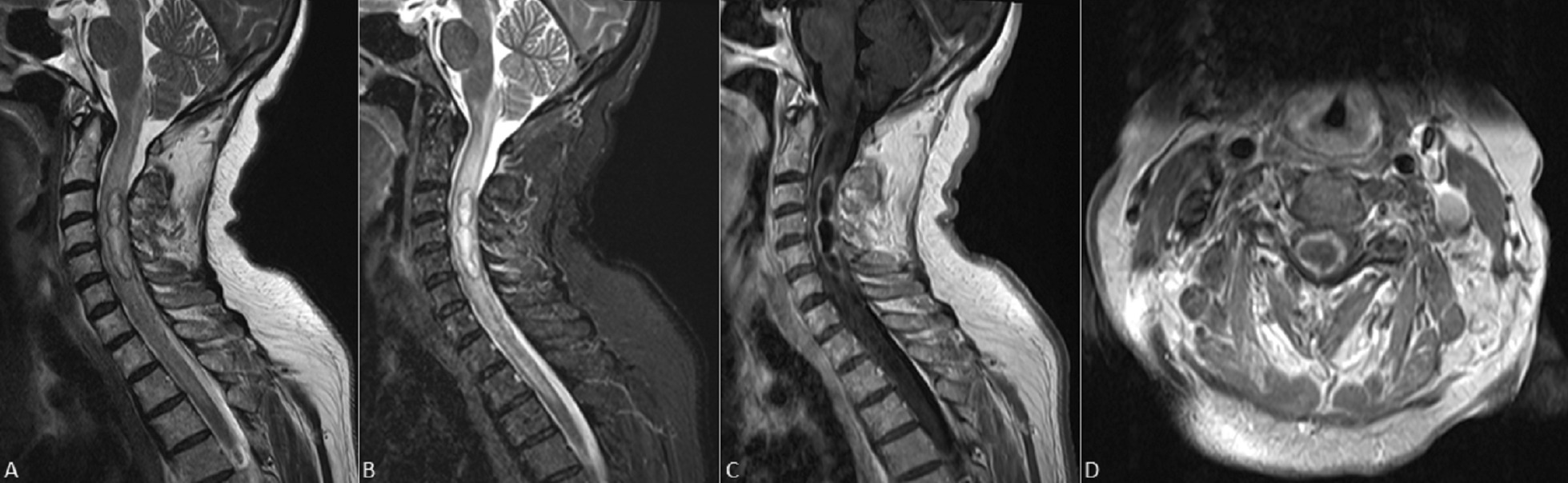
**A** Sagittal T2-weighted and (**B**) short tau inversion recovered cervical M.R. images with an intramedullary hyperintense lesion causing diffuse core expansion and edema. **C** Sagittal and (**D**) axial T1 post-contrast images demonstrate continuous peripheral ring enhancement

On admission, the patient’s exam was notable for weakness throughout her right upper extremity (3/5 strength) and proximal right lower extremity (3/5 strength). The patient was immediately placed on empiric antibiotic therapy with ceftriaxone, vancomycin, and metronidazole; plasma exchange therapy was also initiated given concern for a demyelinating disorder. The neurosurgery service was consulted and a new MRI with diffusion-weighted imaging (DWI) and vascular sequences was obtained, revealing an interval increase in the spinal cord lesion’s size to ~ 41 mm with an associated rim on DWI sequences and extensive cord edema (Fig. 1A–D). Ampicillin was therefore added for coverage of *Listeria* species, and plasma exchange and steroid therapy were discontinued.

The patient underwent emergent surgery (C2–C7 laminectomies followed by a durotomy and a midline myelotomy). After splitting the dorsal columns at the C4–5 level, purulent material was encountered in the middle of the spinal cord and carefully drained using suction irrigation. There was no apparent capsule. After thorough drainage of the abscess, the dura was closed in a watertight fashion.

Immediately following the washout, the patient experienced transient worsening of her right-sided weakness that resolved by post-operative day 1. Operative cultures ultimately grew pan-sensitive *Streptococcus anginosus*, and the antibiotic regimen was narrowed to penicillin G and metronidazole. However, she subsequently developed worsening leukocytosis, and a surveillance MRI on post-operative day 6 revealed interval re-accumulation of fluid and expansion of the ring-enhancing lesion, which now spanned from the cervicomedullary junction to the level of T1 (~ 11 cm). Antibiotics were re-broadened to vancomycin and meropenem. The patient was taken back to the operating room for a second abscess drainage/washout procedure. The laminectomies were extended from C1-7, followed by a midline myelotomy from the cervicomedullary junction to C7. After splitting the dorsal columns purulent material was encountered and carefully drained using suction irrigation. No obvious capsule was identified, though some very friable parenchymal tissue was noted. Fluid was collected and sent for Gram stain and culture. Further drainage and irrigation of purulent material was performed by carefully manipulating the cord to push the purulent material out of the opening. The dura was then closed in a standard fashion. Postoperative MRI showed the abscess wall had coapted, and associated T2 prolongation and expansion of the cord consistent with myelitis. Blood cultures obtained prior to the receipt of antibiotics were negative. Chest, abdomen and pelvic CT scans and transthoracic echocardiogram showed no potential foci of infection. The patient had no history of diabetes, immunodeficiencies and was taking no immunomodulatory medications. Cultures from the second surgical drainage were sterile. Combined antimicrobial therapy with vancomycin and meropenem was continued for five more days, after which vancomycin was discontinued. The patient’s exam was stable post-operatively, with complete paralysis of her right upper extremity (0/5 strength), weakness of her right lower extremity (3/5 strength), and full strength in her left upper and lower extremities. Ultimately the patient was discharged to acute inpatient rehabilitation on IV meropenem for a planned 6 week course. Three weeks into her course she developed eosinophilia and rash and was changed to oral linezolid for the remaining 3 weeks of her course. A short moxifloxacin tail was added on when repeat MRI towards the end of therapy showed a small residual focus of infection.

At 16-month follow-up, the patient’s exam was stable, with complete paralysis of her right upper extremity (0/5 strength) and weakness of her right lower extremity (3/5 strength). The patient uses a manual wheelchair for mobility and a cane for ambulating short distances at home and requires minimal to moderate assistance with activities of daily living (modified Rankin scale 3/6, Nurick grade 4/5).

### Case 2

A 72-year-old man with a history of arthritis, hypertension, deep venous thrombosis, and remote *Staphylococcal aureus* bacteremia was transferred to our institution from an outside hospital after developing acute left lower extremity weakness.

Four days before admission, the patient developed right-sided abdominal pain. He presented to an outside hospital where an abdominal computed tomography (CT) scan was negative for any explanatory lesions. The patient was discharged home only to later represent with complaints of continued lower back and right lower extremity weakness. While undergoing evaluation at the outside hospital, the patient developed new onset left lower extremity weakness (0/5). A spinal MRI was obtained, demonstrating an area of intrinsic T2 hyperintensity from C7 to L1, with associated intradural extramedullary serpiginous flow voids. A preliminary diagnosis of a ruptured spinal arteriovenous malformation (AVM) was made, and the patient was transferred to our institution for emergent angiography and definitive management.

On admission to our institution, the patient endorsed complete loss of sensation beginning at his sternum. His review of systems was otherwise negative, including for constitutional symptoms such as fevers, chills, or night sweats. His physical exam on admission was notable for full strength (5/5) in both upper extremities and flaccid paralysis (0/5) in both lower extremities, with loss of sensation from ~ the T7 dermatome distally; rectal tone was absent.

**Fig. 2:**
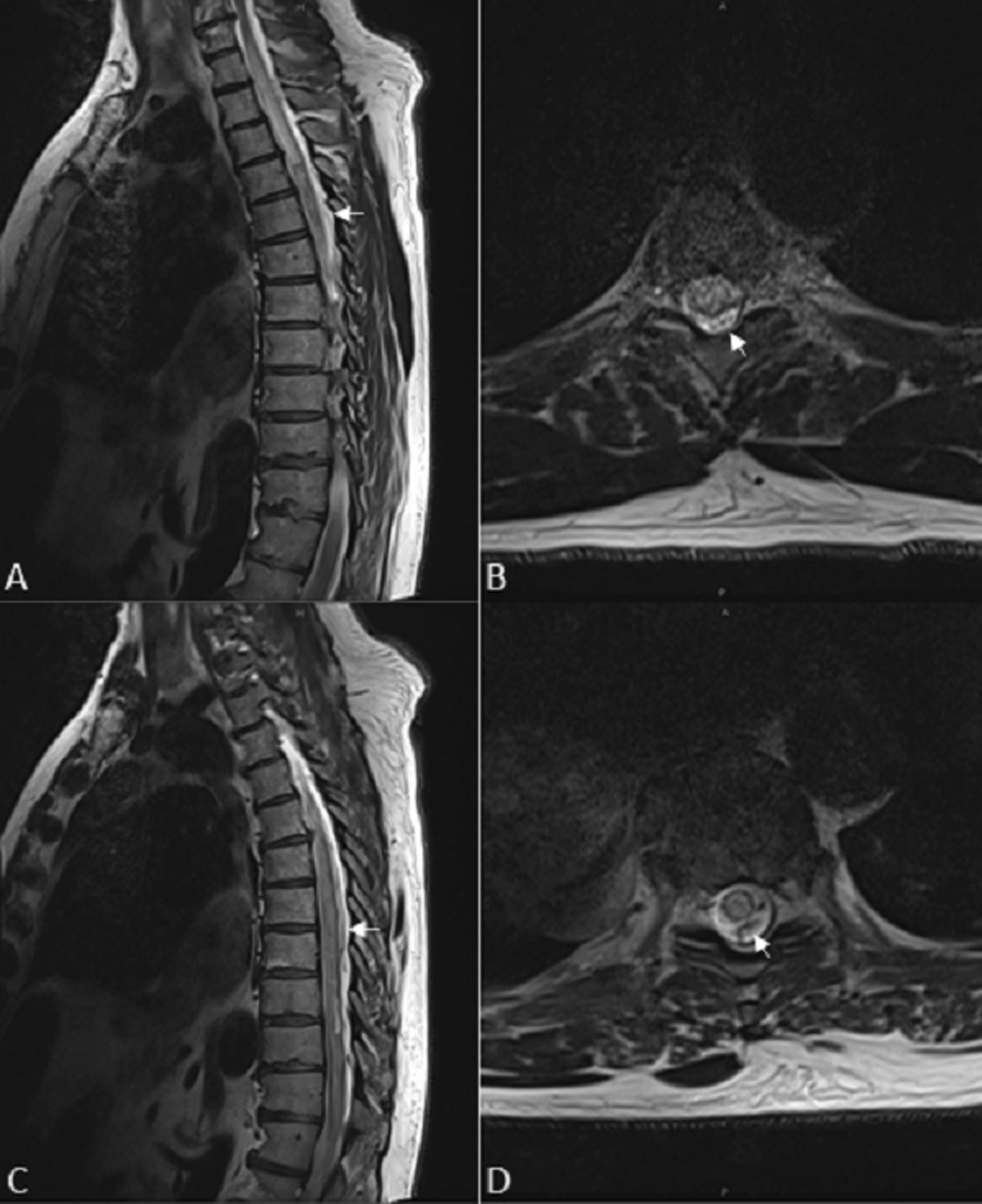
**A** Sagittal and (**B**) axial T2-weighted MR images of the cervicothoracic and (**C**, **D**) lower thoracic spine demonstrates diffuse cord expansion and heterogeneous hyperintensity from C7 to T8 with the severely dorsal-predominant serpiginous flow voids (arrows)

We placed a lumbar drain and obtained straw-colored CSF that was sent for analysis. A spinal angiogram was performed in the interim, which was negative for any vascular lesions/malformations. A repeat contrast-enhanced MRI of the cervical, thoracic, and lumbar spine revealed a peripherally enhancing intramedullary lesion from C7 to L1 (Fig. 2A–D). Resultant CSF analyses revealed a high WBC count at > 700 per µL with a predominance of neutrophils (91%), a low glucose concentration at 20 mg/dL, and a high protein level 359 mg/dL, supporting the diagnosis of an intramedullary abscess. We immediately took the patient to the operating room for decompression and abscess drainage. Given that the abscess was concentrated around T6–7, we performed a focused T6–7 laminectomy and myelotomy to drain the abscess and obtain material for culture. Upon our midline myelotomy, we immediately encountered thick yellowish purulent fluid within the intramedullary cavity and the abscess was drained using gentle irrigation and suction.

**Fig. 3:**
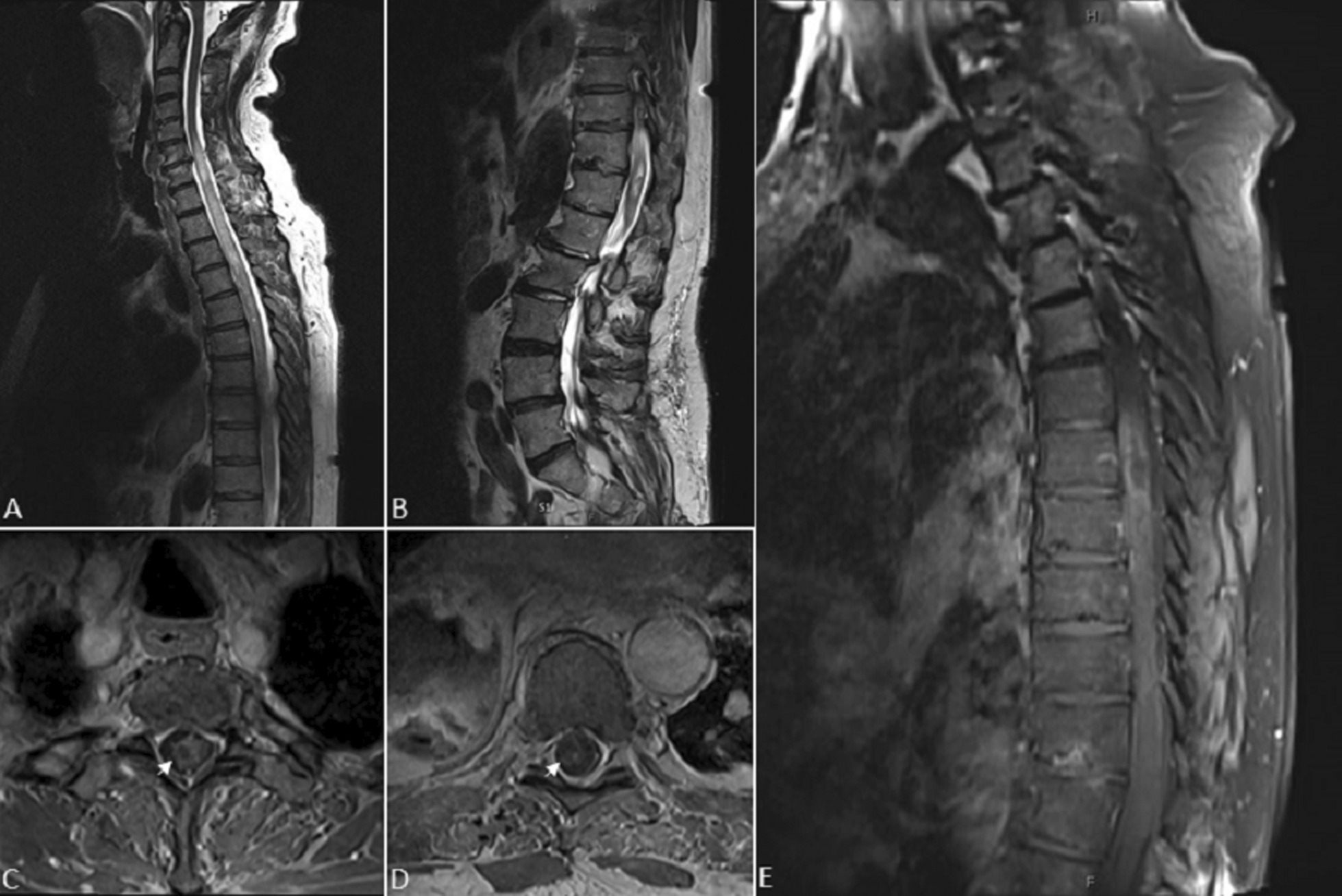
**A** Sagittal cervicothoracic and (**B**) lumbar spine T2-weighted MR images obtained several hours later with progression of expansile heterogeneous hyperintensity now extending from C5 to the conus medullaris. Axial T1 post-contrast images of the (**C**) upper and (**D**) lower thoracic spine demonstrate foci of ring enhancement within the right hemi-cord (arrows). **E** The peripheral ring-enhancement is also visible on sagittal T1 post-contrast images

Post-operatively the patient was started on empiric ceftriaxone, vancomycin, ampicillin, and metronidazole. Intraoperative abscess cultures eventually grew out penicillin-sensitive *Streptococcus anginosus* and the patient’s antibiotic regimen was narrowed to vancomycin and ceftriaxone. Approximately 12 h after the index operation, the patient declined clinically and developed right-hand weakness. A repeat MRI revealed cranial extension of the spinal cord abscess to the level of C5 (Fig. 3A–E). As such, the patient was immediately taken back to the operating room for an emergent C7–T1 laminectomies and repeat intramedullary abscess evacuation.

Evaluation for a primary source was notable for negative blood cultures (drawn prior to the administration of antibiotics), a transthoracic echocardiogram without evidence of valvular vegetations, and no evidence of odontogenic infection on exam or history. He had no history of diabetes or other immunosuppressing conditions, no history of intravenous drug use, and was not taking steroids or other immunosuppression at the time of presentation.

**Fig. 4:**
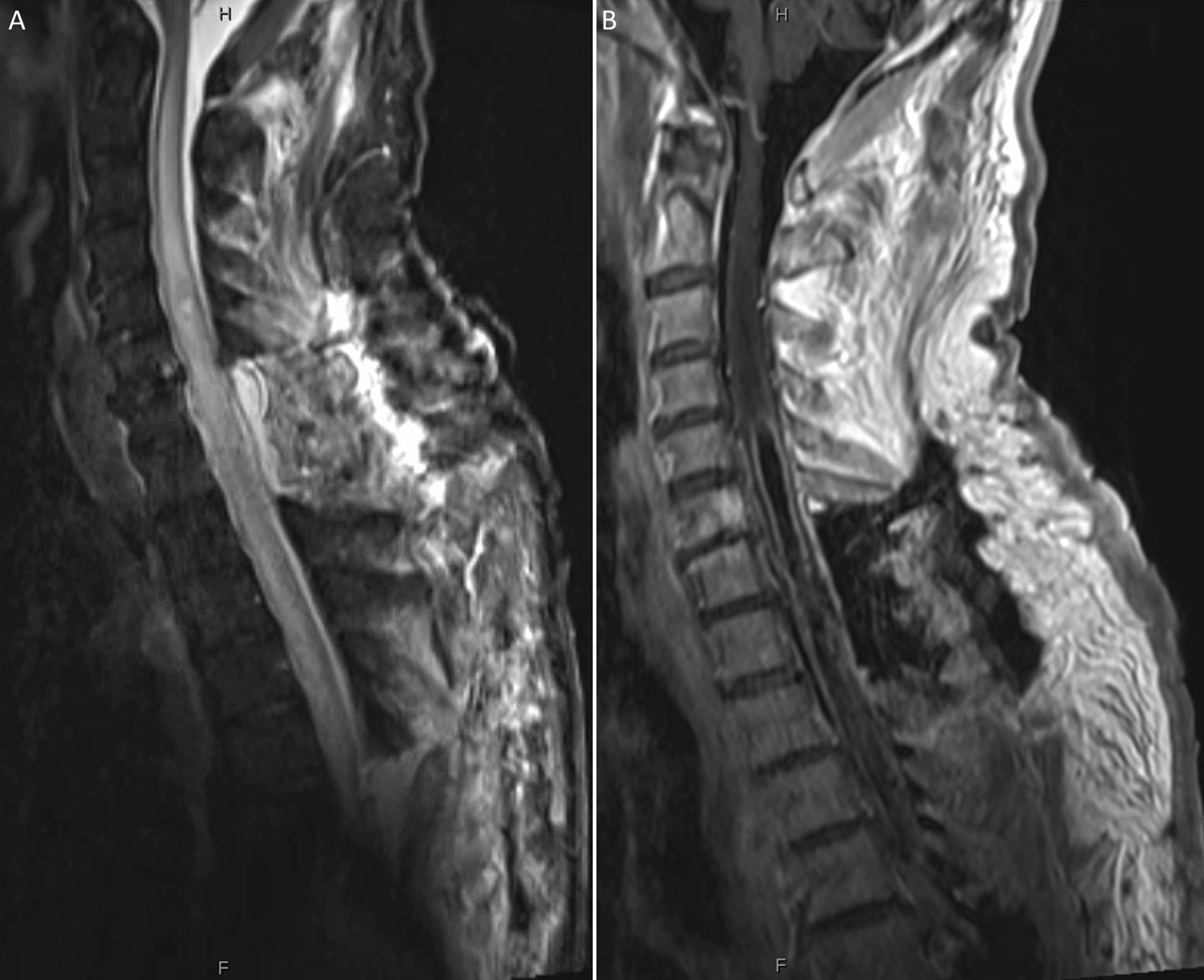
**A** Sagittal cervicothoracic STIR and (**B**) T1 post-contrast MR obtained on postoperative day 4 demonstrate a thinner rind of enhancement consistent with improving, but persistent, infection as well as admixed inflammatory and postoperative edema

On post-operative day 4, a contrast-enhanced MRI demonstrated decreased, but persistent, rim-enhancement and a mixture of inflammatory and postoperative edema (Fig. 4). The patient’s clinical course was stable. He was discharged to an acute inpatient rehab facility on post-operative day 8 on a 6 week course of IV penicillin G.

At 4-week follow-up, the patient’s exam was noted to be stable, with weakness of his upper extremities right (3/5 strength) greater than left (4/5 strength) and complete paralysis of his lower extremities (0/5 strength). The patient uses a wheelchair for mobility and requires moderate to maximal assistance with activities of daily living (modified Rankin scale 4/6, Nurick grade 5/5).

## Discussion and conclusion

Intramedullary abscesses are rare infections. Here we have described two such cases illustrating challenges with both initial diagnosis and definitive clinical management. Given the aggressive nature of such infections, prompt recognition and source control are essential. It is prudent to note that both of our cases ultimately required re-operation/repeat drainage despite the initiation of appropriate antimicrobial therapy (i.e., as evidenced by negative repeat operative cultures). Over 125 cases of intramedullary abscesses have previously been reported in the literature [12], including five previous cases caused by *Streptococcus anginosus* group organisms [13–17]. Interestingly, none of these cases reported flow voids on pre-operative MRI, making our second case the first to highlight such lesions’ potential to mimic a spinal AVM.

Intramedullary infections can be precipitated by several risk factors, including dermoid cysts [18]. intravenous drug use [6], genitourinary infections [1], osteomyelitis [19], penetrating or iatrogenic injuries to the spine [20], endocarditis [2], septic embolism [7], bacterial meningitis [8], and pre-existing spinal pathologies, including spinal tumors, AVMs or dural arteriovenous fistulas [21, 22]. Of note, the patients reported here did not appear to have any of these predisposing conditions. *Streptococcus anginosus* group bacteria are gram-positive organisms and part of the microbiome of the oral cavity and gastrointestinal tract [23]. They are common pathogens in abscesses, and as demonstrated in this report and via previously reported cases, there are often no obvious sources and/or predisposing factors for infection [13–15]. It is thought these bacteria produce cytolytic toxins and other virulence factors that lead to tissue damage and abscess formation [23, 24].

*Mycobacterium tuberculosis* is one of the primary causes of intramedullary abscesses in the developing world, with numerous cases having been reported [25]. Outside of the developing world, gram-positive cocci, especially those native to oral or skin flora (as were the cases here), are common causes of intramedullary abscesses [11, 14, 15, 26–31]. Opportunistic pathogens such as *Fusarium* spp. or *Aspergillus* spp. have also been reported as a cause of intramedullary abscesses in immunocompromised hosts, which has implications for the selection of an empiric antimicrobial regimen in this population and further underscores the importance of obtaining culture data to direct therapy [32, 33]. As such, clinicians should consider the potential wide array of pathogens capable of causing intramedullary abscesses, including the patient’s risk for tuberculosis, fungal infection and immune status, when starting broad empiric coverage. Based on reported microbiological data, a reasonable empiric regimen in an immunocompetent patient without recent instrumentation would be vancomycin, ceftriaxone, and metronidazole, which treats gram positive bacteria (including methicillin-resistant *Staphylococcus aureus*), gram negative bacteria, and anaerobes. The antimicrobials in this regimen have penetration into the central nervous system (CNS) near the mean inhibitory concentration of moderately susceptible bacteria even in the absence of significant meningeal inflammation [34].

Clinically, intramedullary abscesses can present with acute neurological deficits, mimicking an episode of transverse myelitis [35]. More commonly, intramedullary abscesses have a presentation characterized by progressive dorsal pain followed by neurological deficits as was seen in our patients [14, 29, 36, 37]. On occasion, the clinical presentation can be more insidious, mimicking a spinal tumor or other conditions capable of inducing chronic myelopathy [38]. Multifocality of abscesses can further complicate an already complex clinical picture [39]. Finally, recurrent meningitis may also result from the rupture of such abscesses into the subarachnoid space [40, 41].

Concerning imaging, when an intramedullary abscess is suspected, contrast enhanced MRI is the primary modality of choice. Intramedullary abscesses are typically hyperintense on T2-weighted images and enhance peripherally on contrast-enhanced T1-weighted scans [13]. As infection resolves following treatment, hyperintensity on T2-weighted images tends to resolve [13]. A precipitation or drop sign—characterized by an accumulation of pus at the conus medullaris—has also been previously described and may be more specific for spinal abscess [42].

Regarding treatment, the most common therapeutic approaches to intramedullary abscesses center on surgical decompression and myelotomy for abscess drainage with cavity content sampling for culture-guided antibiotic therapy yet reports of intramedullary abscesses managed non-operatively do exist [36, 43]. For previously described intra-medullary abscesses caused by *Streptococcus* species, beta-lactams such as penicillin and ceftriaxone have been common antibiotic choices due to their excellent anti-streptococcal activity and CNS penetration [13–17]. The two cases described here received distinct individualized antimicrobial regimens based on their clinical course. In the first case, the patient was broadened to meropenem in the setting of clinical decompensation, in order to treat both streptococci as well as potential newly-acquired nosocomial pathogens such as *Pseudomonas aeruginosa*. While no additional pathogens were recovered from subsequent cultures, she remained on this regimen initially due to her clinical improvement on meropenem. Several weeks into her course she was changed to linezolid, an oxazolidinone, and then moxifloxacin, a fluoroquinolone, in the setting of concern for a developing allergy to beta-lactams. Both of these agents also have excellent CNS penetration [34]. In the second case, penicillin was ultimately chosen as a narrow spectrum agent for the patient’s penicillin-susceptible streptococcal infection. In terms of duration of therapy, there are no standardized guidelines for treatment of intramedullary abscesses. In our cases, we extrapolated from existing guidelines around the treatment of brain abscesses which recommend a duration of at least 6 weeks following surgical drainage [44]. This course is similar to what has been reported in other cases of intra-medullary abscesses with *Streptococcus* species (anywhere between 6 and 12 weeks) [13–17].

In conclusion, we report two cases of intramedullary abscesses without known predisposing factors or primary infections caused by *Streptococcus anginosus*. Both cases presented with dorsal midline pain, rapidly progressive myelopathy, and muscle weakness. Delayed diagnosis occurred in both instances before transferring to our institution due to this condition’s rarity and propensity to mimic other pathologies. Critically, each case was treated surgically and required repeat abscess drainage in addition to prolonged antibiotic treatment.

The authors’ position is that while rare, it is critical to consider intramedullary abscesses on the differential for any MRI lesions that are hyperintense on T2 and peripherally enhancing on contrast enhanced T1 sequences, thereby minimizing time to appropriate and definitive treatment.

## Data Availability

Not applicable.

## Abbreviations

MRI: Magnetic resonance imaging
WBC: White blood cells
DWI: Diffusion-weighted imaging
CT: Computed tomography
AVM: Arteriovenous malformation
CSF: Cerebrospinal fluid
STIR: Short tau inversion recovery
